# Q1291H-CFTR molecular dynamics simulations and *ex vivo* theratyping in nasal epithelial models and clinical response to elexacaftor/tezacaftor/ivacaftor in a Q1291H/F508del patient

**DOI:** 10.3389/fmolb.2023.1148501

**Published:** 2023-06-01

**Authors:** Katelin M. Allan, Miro A. Astore, Egi Kardia, Sharon L. Wong, Laura K. Fawcett, Jessica L. Bell, Simone Visser, Po-Chia Chen, Renate Griffith, Adam Jaffe, Sheila Sivam, Orazio Vittorio, Serdar Kuyucak, Shafagh A. Waters

**Affiliations:** ^1^ School of Clinical Medicine, Discipline of Paediatrics and Child Health, Faculty of Medicine and Health, UNSW Sydney, Sydney, NSW, Australia; ^2^ Molecular and Integrative Cystic Fibrosis Research Centre, UNSW Sydney, Sydney, NSW, Australia; ^3^ School of Biomedical Sciences, Faculty of Medicine and Health, UNSW Sydney, Sydney, NSW, Australia; ^4^ School of Physics, The University of Sydney, Sydney, NSW, Australia; ^5^ Department of Respiratory Medicine, Sydney Children’s Hospital, Sydney, NSW, Australia; ^6^ Children’s Cancer Institute, UNSW Sydney, Sydney, NSW, Australia; ^7^ Department of Respiratory Medicine, Royal Prince Alfred Hospital, Sydney, NSW, Australia; ^8^ School of Natural Sciences (Chemistry), University of Tasmania, Hobart, TAS, Australia

**Keywords:** cystic fibrosis, CFTR, modulators, airway epithelial cell models, personalized medicine, molecular dynamics, c.3873G>C

## Abstract

**Background:** Cystic fibrosis (CF) is caused by a wide spectrum of mutations in the CF transmembrane conductance regulator (*CFTR*) gene, with some leading to non-classical clinical presentations. We present an integrated *in vivo, in silico* and *in vitro* investigation of an individual with CF carrying the rare Q1291H*-CFTR* allele and the common F508del allele. At age 56 years, the participant had obstructive lung disease and bronchiectasis, qualifying for Elexacaftor/Tezacaftor/Ivacaftor (ETI) CFTR modulator treatment due to their F508del allele. Q1291H *CFTR* incurs a splicing defect, producing both a normally spliced but mutant mRNA isoform and a misspliced isoform with a premature termination codon, causing nonsense mediated decay. The effectiveness of ETI in restoring Q1291H-CFTR is largely unknown.

**Methods:** We collected clinical endpoint measurements, including forced expiratory volume in 1 s percent predicted (FEV1pp) and body mass index (BMI), and examined medical history. *In silico* simulations of the Q1291H-CFTR were compared to Q1291R, G551D, and wild-type (WT)-CFTR. We quantified relative Q1291H *CFTR* mRNA isoform abundance in patient-derived nasal epithelial cells. Differentiated pseudostratified airway epithelial cell models at air liquid interface were created and ETI treatment impact on CFTR was assessed by electrophysiology assays and Western blot.

**Results:** The participant ceased ETI treatment after 3 months due to adverse events and no improvement in FEV1pp or BMI. *In silico* simulations of Q1291H-CFTR identified impairment of ATP binding similar to known gating mutants Q1291R and G551D-CFTR. Q1291H and F508del mRNA transcripts composed 32.91% and 67.09% of total mRNA respectively, indicating 50.94% of Q1291H mRNA was misspliced and degraded. Mature Q1291H-CFTR protein expression was reduced (3.18% ± 0.60% of WT/WT) and remained unchanged with ETI. Baseline CFTR activity was minimal (3.45 ± 0.25 μA/cm^2^) and not enhanced with ETI (5.73 ± 0.48 μA/cm^2^), aligning with the individual’s clinical evaluation as a non-responder to ETI.

**Conclusion:** The combination of *in silico* simulations and *in vitro* theratyping in patient-derived cell models can effectively assess CFTR modulator efficacy for individuals with non-classical CF manifestations or rare *CFTR* mutations, guiding personalized treatment strategies and optimizing clinical outcomes.

## 1 Introduction

Cystic fibrosis (CF) is a monogenic disease caused by mutations in the CF transmembrane conductance regulator (*CFTR*) gene that may impair expression, function or stability of the CFTR protein (Elborn, 2016). CFTR is an anion channel which functions at the apical surface of epithelial cells and maintains fluid homeostasis throughout the body, such as in the lungs, pancreas, liver, intestine, and sweat ducts (Rowe et al., 2005).

The tertiary structure of CFTR includes two transmembrane domains (TMD) that form an ion conducting channel pore, two nucleotide binding domains (NBD) that are binding sites for ATP and Mg^2+^, and a regulatory domain (Zhang et al., 2018). ATP and Mg^2+^ binding to the NBDs is necessary for the TMDs to transition into the open conformation and allow the passage of ions. Currently, there are four targeted small molecule drugs, known as CFTR modulators, available to treat patients with CF. CFTR modulators correct CFTR protein dysfunction by two main modes of action: potentiators (e.g., VX-770, ivacaftor (IVA)) increase channel open probability and correctors (e.g., VX-809, lumacaftor (LUM); VX-661, tezacaftor (TEZ); VX-445, elexacaftor (ELX)) rescue misfolded CFTR protein and chaperone it to the cell surface [reviewed in (Lopes-Pacheco, 2019)]. They are used either alone or in combination since a *CFTR* mutation can present several defects (e.g., F508del) (Veit et al., 2016). The triple combination therapy ELX/TEZ/IVA (ETI) is currently the most effective treatment as demonstrated by improvement in patient lung function (Heijerman et al., 2019). It has been approved by the US Food and Drug Administration, Australian Therapeutic Goods Administration and European Medicines Agency for patients with at least one F508del allele regardless of the second allele (Middleton et al., 2019). However, clinical responsiveness to CFTR modulators is variable among those who have access to these treatments (Boyle et al., 2014; Wainwright et al., 2015; Heijerman et al., 2019; Middleton et al., 2019). This adds complexity to predicting treatment response for patients with rare compound heterozygous *CFTR* mutations. Various patient derived cell models [reviewed in (10)] have been shown to replicate the genetic makeup of the individual they were created from. Importantly, CFTR functional testing in patient-derived cell models correlates with the *in vivo* clinical outcomes for that patient (Dekkers et al., 2016; McGarry et al., 2017; Mutyam et al., 2017; McCarthy et al., 2018; Phuan et al., 2021; Terlizzi et al., 2021; Allan et al., 2022; Ciciriello et al., 2022). As such, they are useful tools to predict patient responsiveness to CFTR modulators and characterize rare mutations (Awatade et al., 2018).

**FIGURE 1:**
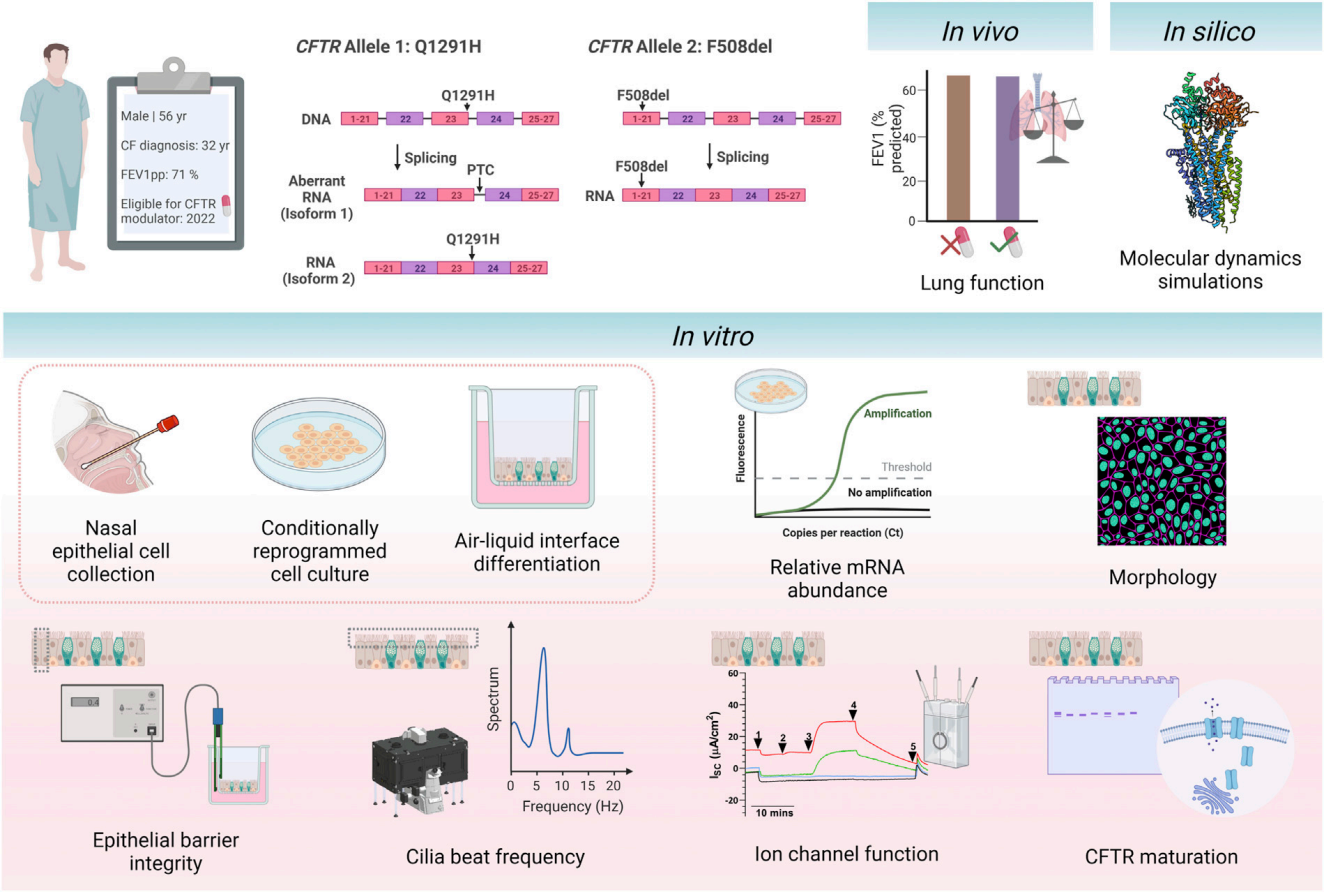
Schematic of study design. A 56-year-old individual with cystic fibrosis with the *CFTR* genotype Q1291H/F508del became eligible for CFTR modulator elexacaftor/tezacaftor/ivacaftor in April 2022 due to their F508del allele. The Q1291H *CFTR* mutation incurs a splicing defect, resulting in misspliced mRNA causing a premature termination codon (isoform 1) and normally spliced mRNA (isoform 2). The F508del *CFTR* mutation results in the deletion of phenylalanine 508. The participant’s *in vivo* clinical outcomes were assessed before and after initiation of treatment with ELX/TEZ/IVA (ETI). *In silico* modelling of the Q1291H-CFTR protein was performed using molecular dynamic simulations to visualise the structural defect occurring in normally spliced protein product. Primary human nasal epithelial cells (hNECs) were collected from the participant prior to commencing treatment with ETI. Relative abundance of Q1291H and F508del *CFTR* mRNA was quantified. hNECs were differentiated at air-liquid interface to form pseudostratified airway epithelial cell models. The following parameters were assessed in differentiated cell models with and without addition of ETI treatment: morphology, epithelial barrier integrity, cilia beat frequency, CFTR maturation and ion channel function. This figure was created with BioRender.com.

Missense mutations within the NBD2 domain of the CFTR protein, wherein glutamine is substituted for a histidine (Q1291H, c.3873G>C) or arginine (Q1291R, c.3872A>G), are currently reported on 39 alleles in the CFTR2 database (Clinical and Functional Translation of Cftr, 2023). While Q1291H is not currently eligible for any CFTR modulator treatment, Q1291R has been approved for treatment—initially for IVA, and later also for TEZ/IVA and ETI [CFTR modulator approvals reviewed in (Lopes-Pacheco et al., 2021; Costa et al., 2022)]. While Q1291H has not been formally classified, it has been shown to lead to aberrant splicing resulting in formation of two CFTR mRNA isoforms (Jones et al., 1992; Joynt et al., 2020) (Figure 1). Isoform 1 is an aberrantly spliced RNA transcript that produces a premature termination codon (PTC) and nonsense-mediated mRNA decay (NMD) thus no protein product is formed. Isoform 2 is normally spliced and results in a protein with the point mutation Q1291H. The proportion of aberrantly spliced and normally spliced CFTR transcript varies between individuals [reviewed in (Castellani et al., 2008)], which in turn impacts the amount of residual CFTR protein function, and thus disease severity (Nissim-Rafinia et al., 2004). In a heterologous expression system where Q1291H (c.3873G>C cDNA, i.e., isoform 2) was expressed in CF bronchial epithelial cells (CFBEs) lacking endogenous *CFTR* expression (CFBE41o-), CFTR-specific current measured at 80% of wild-type (WT) CFTR, which was increased by IVA (1.9-fold), LUM (1.4-fold), and LUM/IVA (2.2-fold) (Joynt et al., 2020). However, cDNA-based expression systems cannot account for aberrantly spliced product and therefore, may misrepresent the baseline CFTR function and potential response to modulator *in vivo.*

This study assessed clinical response to ETI *in vivo* in a 56-year-old individual with CF with the Q1291H/F508del *CFTR* genotype (Figure 1). While remaining blind to the participant’s clinical response to ETI, using MD simulations, we determined the structural defect of CFTR protein resulting from the normally spliced Q1291H isoform. We also assessed the molecular, functional and biochemical consequence of the Q1291H-CFTR mutation using primary human nasal epithelial cell (hNEC) models derived from the above participant and compared the *in vitro* results with the *in vivo* clinical response of the patient.

## 2 Materials and methods

### 2.1 Recruitment and clinical data collection

Written informed consent was obtained from participants with Q1291H/F508del (*n* = 1), F508del/F508del (*n* = 3), G551D/F508del (*n* = 3) and WT/WT (*n* = 1) *CFTR* genotypes (Supplementary Table S1) or the participants’ guardian/parents, with approval from the Sydney Children’s Hospital Ethics Review Board (HREC/16/SCHN/120). The participants’ nasal inferior turbinate was brushed, and human nasal epithelial cells (hNECs) were collected as previously described (Allan et al., 2021). Participants were not on any CFTR modulators at the time of sample collection. Medical records of the participant with Q1291H/F508del *CFTR* genotype were reviewed to collect medical history and clinical endpoints of FEV1 and BMI. Information about this participant’s childhood presentation and route to CF diagnosis has previously been reported in the literature (Wellesley et al., 1998).

### 2.2 Molecular dynamics simulations

The simulation methodologies for this study are the same as those used previously (Allan et al., 2022; Wong et al., 2022a; Wong et al., 2022b). In brief, an extended model of the CFTR protein bound to ATP under phosphorylating conditions (PDB ID: 6MSM) was embedded in a 1-palmitoyl-2-oleoyl-sn-glycero-3-phosphocholine (POPC) model bilayer then solvated with a solution containing 150 mmol KCl. The construction of the extended model is detailed in a previous publication (Wong et al., 2022a). The model of Q1291H-CFTR was constructed with the H1291 sidechain as the neutral Nδ tautomer. The WT-CFTR system consisted of 207 potassium ions, 218 chloride ions, 236 POPC molecules and 45,458 water molecules. In all simulated systems, the balance of ions was changed to ensure that the overall system was electrically neutral.

Gromacs 2021.1 (Abraham et al., 2015) was used for all MD simulations with the CHARMM36m force field (Huang et al., 2017). To improve performance by setting a simulation timestep of 4 femtoseconds, virtual site topologies have been employed for all non-water molecules: CFTR protein (Feenstra et al., 1999), ATP molecules as generated via MkVSite (Larsson et al., 2020), and POPC lipids (Olesen et al., 2018). Periodic boundary conditions were enforced, with long range electrostatic potentials calculated using the Particle Mesh Ewald (PME) summation with a cutoff of 12 Å (Essmann et al., 1995). A Nosé-Hoover thermostat and a Parrinello-Rahmann barostat were used for all simulations to maintain constant temperature and pressure at 310 K (37°C) and 1 atm, respectively (Parrinello and Rahman, 1981; Nosé and Klein, 1983; Berendsen et al., 1984). Relaxation time parameters for the thermostat and barostat were 1 and 5 picoseconds respectively.

Three replicates were generated for each mutant, starting from energy minimization as described below. The initial positions of all molecules were equilibrated for production runs in two phases: first via energy minimization using a steepest descent algorithm, then followed by a 6 ns simulation where harmonic position-restraints on all non-hydrogen atoms were imposed at 10 kcal/mol/Å^2^ and then gradually relaxed. The magnitude of these restraints were halved every 400 picoseconds for 15 iterations. Another 10 nanoseconds of unrestrained equilibration followed this restrained period. This process was followed by a production run of 2 microseconds. Scripts may be accessed at https://github.com/miro-astore/gromacs_scripts, while simulation files are available upon request.

### 2.3 Participant nasal epithelial cell expansion

Collected cells were seeded in collagen I (Advanced Biomatrix 5015) coated vessels pre-seeded with irradiated NIH/3T3 feeder cells (irrNIH/3T3) and expanded via the conditional reprogramming cell (CRC) method as previously described (Allan et al., 2021). At 90% confluence, hNECs were dissociated via differential trypsinisation and were cryopreserved.

### 2.4 Real-time quantitative PCR (qPCR)

hNECs from the Q1291H/F508del participant and three F508del/F508del participants were homogenized with 0.5 mL of cold TRIzol (Thermo Fisher Scientific, Waltham, MA) for RNA extraction. RNA purification was performed with a RNeasy Mini Kit (QIAGEN, Hilden, Germany) following the manufacturer’s protocol with an on-column RNase-free DNase Set (QIAGEN, Hilden, Germany) treatment. RNA was quantified and for each sample, 120 ng of RNA was used as input for cDNA preparation using the M-MLV Reverse Transcriptase and random hexamer priming. Phe508del and non-F508del *CFTR* was detected by RT-qPCR (in technical triplicate) using the CFX384 Touch Real-Time PCR Detection System (Bio-Rad, Hercules, CA) with primer pairs from Clarke et al. (2019) (Clarke et al., 2019): non-F508del CFTR Forward: GGC​ACC​ATT​AAA​GAA​AAT​ATC​ATC​TT; Reverse: CTT​GCT​CGT​TGA​CCT​CCA​CT). Phe508del CFTR Forward: GGC​ACC​ATT​AAA​GAA​AAT​ATC​ATT​GG and Reverse: CTT​GCT​CGT​TGA​CCT​CCA​CT. CT values were normalized to the reference gene ACTB (*β* actin) (F: AGA​AAA​TCT​GGC​ACC​ACA​CC; R: AGA​GGC​GTA​CAG​GGA​TAG​CA.). As described previously (Clarke et al., 2019), products were quantified using the ΔΔCT method. In brief, mRNA expression was first normalized using ACTB. To calculate the ΔΔCT, the ΔCT was normalized to the mean ΔCT of the calibrator samples (*n* = 3 F508del/F508del). The fold change (FC) between F508del and non-F508del products was calculated using the formula FC = 2(^–ΔΔCT^). Abundance of *CFTR* transcripts derived from each allele was expressed as a percentage of total *CFTR*. To calculate the percent degradation of non-F508del transcripts, the following formula was used: % degradation = [(Y–X)/Y] × 100, where X and Y are percent *CFTR* transcript derived from non-F508del and F508del alleles, respectively, assuming 50% transcript from each allele in the absence of a splicing defect resulting in degradation.

### 2.5 Differentiated airway epithelia at air-liquid interface (ALI)

Collagen I coated Transwell porous polyester membranes (6.5 mm 0.4 µm, Sigma CLS3470) were seeded with passage two hNECs (250,000 cells/membrane) in PneumaCult Ex Plus expansion media (STEMCELL Technologies 05040). Once a confluent monolayer was reached, the medium was changed to PneumaCult ALI medium (STEMCELL Technologies 05001) and hNECs were differentiated to create air-liquid interface (ALI) epithelia as described previously (Allan et al., 2021; Awatade et al., 2021; Wong et al., 2022b) by removal of media from the apical compartment. Media in the basal compartment were changed every second day for 18–21 days until a mature pseudostratified epithelium with beating cilia was established. Differentiated hNECs with transepithelial electrical resistance (TEER) readings within the range previously reported in airway cell models at ALI (188–1250 Ω cm^2^) (Wong et al., 2022b) were considered mature.

### 2.6 Treatment of differentiated airway epithelia with CFTR modulator

Pseudostratified differentiated hNECs were incubated (basal side) with 3 μM VX-445 (ELX, Selleckchem S1565) and 18 μM VX-661 (TEZ, Selleckchem S7059) as per concentrations previously published (Keating et al., 2018), or vehicle control (0.01% DMSO), for 48 h prior to experiments. At 48 h following treatment, cilia beat frequency (CBF) was measured (section 2.8) in differentiated hNECs prior to electrophysiological assessment of CFTR-mediated ion transport (Section 2.9). 10 μM VX-770 (IVA, Selleckchem S1144) or 0.01% DMSO was added acutely to the apical compartment of the Ussing chamber during CFTR-mediated ion transport assays. Post electrophysiological assessment treated differentiated hNECs were used for immunofluorescence (Section 2.7) and Western blotting (Section 2.10).

### 2.7 Immunofluorescence

Differentiated hNECs were processed as previously described (Wong et al., 2022c). Briefly, samples were fixed in 4% paraformaldehyde for 15 min at room temperature. Fixed samples were permeabilized with 0.5% Triton-X in PBS for 10 min on ice and blocked using IF buffer (0.1% BSA, 0.2% Triton and 0.05% Tween 20 in PBS) with 10% normal goat serum (Sigma G9023) for 1 h at room temperature. Samples were incubated with Acetyl-α-Tubulin (Lys40) (D20G3) XP^®^ Rabbit mAb (Alexa Fluor^®^ 647 Conjugate) (Cell Signalling 81502S), Phalloidin-Atto 565 (Sigma 94072) and DAPI (ThermoFisher D1306) for 3 h at room temperature. Membranes were excised from transwells and mounted on SuperFrost Plus slides (Thermo Fisher Scientific, Waltham, MA) with Vectashield Plus antifade mounting medium (H-1900; Vector Laboratories, Burlingame, CA). Images were acquired using a Leica TCS SP8 DLS confocal microscope (Leica Microsystems, Wetzlar, Germany) with a 63x/1.4 oil immersion objective. Image processing was performed using ImageJ software (National Institute of Health, Bethesda, MD).

### 2.8 Quantification of cilia beat frequency

Cilia beating in differentiated hNECs was imaged and analysed as previously described (Allan et al., 2021). Imaging was performed using a Nikon Eclipse Ti2-E microscope with an Andor Zyla 4.2 sCMOS camera and a CFI S Plan Fluor ELWD 20×/0.45 objective. Time-lapse images were acquired from six fields of view from each of *n* = 2–3 differentiated hNEC cultures per participant. Cilia beat frequency (CBF) was analysed using a custom-built script in Matlab (MathWorks, Natick, MA) that identified the dominant frequency (highest peak).

### 2.9 Quantification of CFTR-mediated ion transport

Differentiated hNECs were mounted in circulating Ussing chambers (VCC MC8; Physiologic Instruments, San Diego, CA). Chambers were filled with asymmetric chloride (Cl^−^) Ringer solution (bicarbonate free) and short-circuit current (*I*_sc_, µA/cm^2^) was measured as previously described (Awatade et al., 2021). Baseline current was stabilised for 30 min before transepithelial electrical resistance (TEER) measurements were recorded. The following pharmacological compounds were then sequentially added: 100 μM apical amiloride to inhibit epithelial sodium channels, 10 μM apical VX-770 (IVA) or 0.01% DMSO (vehicle) to potentiate CFTR-activated currents, 10 μM basal forskolin to induce cAMP activation of CFTR, 30 μM apical CFTR_Inh-172_ to inhibit CFTR-specific currents and 100 μM apical ATP to activate calcium-activated chloride currents. Using Acquire and Analysis 2.3 software (Physiologic Instruments, San Diego, CA) data recordings of *I*_sc_ were obtained. Cumulative changes of *I*_sc_ in response to forskolin and CFTR modulator were used to quantify total CFTR-activated currents. Changes in *I*_sc_ in response to forskolin alone (no CFTR modulator) were considered baseline activity (Δ*I*_sc-Fsk_).

### 2.10 Western blotting

Differentiated hNECs were lysed with TNI lysis buffer (0.5% Igepal CA-630, 50 mM Tris pH 7.5, 250 mM NaCl, 1 mM EDTA) (Pankow et al., 2015) containing protease inhibitor cocktail (Roche 04693159001) for 30 min on ice. Lysates were sonicated using the Bioruptor Pico (Diagenode, Liège, Belgium) as previously described (Wong et al., 2022b). Lysate protein concentration was measured using a BCA Protein Assay Kit (Thermo Fisher Scientific 23225). Lysates were separated using a NuPAGE 3%–8% Tris-Acetate gel (Thermo Fisher Scientific EA0375BOX) at 100 V for 30 min, and then at 150 V until separation was complete. To avoid signal saturation variable amounts of protein were added per sample (WT/WT = 20 μg, Q1291H/F508del = 40 μg, F508del/F508del = 100 μg). Proteins were transferred onto a nitrocellulose membrane via wet transfer at 20 V for 1 h. CFTR was detected with anti-CFTR antibody 596 (1:500; University of North Carolina, Chapel Hill and Cystic Fibrosis Foundation) at 4°C overnight. Protein bands were visualized using ECL Select detection reagent (Cytiva RPN2235) on an ImageQuant LAS 4000 (GE Healthcare, Chicago, IL). Anti-calnexin antibody (1:1000; Cell Signalling Technology 2679) was used to detect calnexin loading control. Protein band densitometry was conducted using ImageJ (National Institutes of Health, Bethesda, MD). Data were normalized to the calnexin loading control.

### 2.11 Statistical analysis

Statistical analysis advice was received from Stats Central, UNSW. The normality of data distribution was assessed using the Shapiro-Wilk test. For normally distributed data, an ordinary one-way ANOVA with Tukey’s method for multiple comparisons or an unpaired *t*-test was used to determine statistical significance as indicated in the figure legends. For data that did not follow a normal distribution (Supplementary Figures S1A–C), the Kruskal-Wallis non-parametric test was applied. All statistical analysis was performed using GraphPad Prism v9.0.1 (GraphPad Software, San Diego, CA). Data are presented as mean ± standard error of the mean (SEM). A *p*-value of <0.05 was considered statistically significant.

## 3 Results

### 3.1 Clinical response to treatment with ETI

The participant was managed for severe asthma from age 6, with no pancreatic disorders and a sweat chloride test result which was normal (<40 mmol/L normal range for children at the time of testing). As such, CF was considered unlikely. *CFTR* genotyping of the individual, identified heterozygous F508del and Q1291H alleles, leading to CF diagnosis at age 32 (Wellesley et al., 1998). By adulthood our patient had significant impairment in lung function (forced expiratory volume in 1 s (FEV1): 71% predicted) and bronchiectatic changes visible on his chest x-ray (additional clinical history in Supplementary Material S1). The participant became eligible for treatment with ETI in April 2022 due to his F508del allele.

**FIGURE 2:**
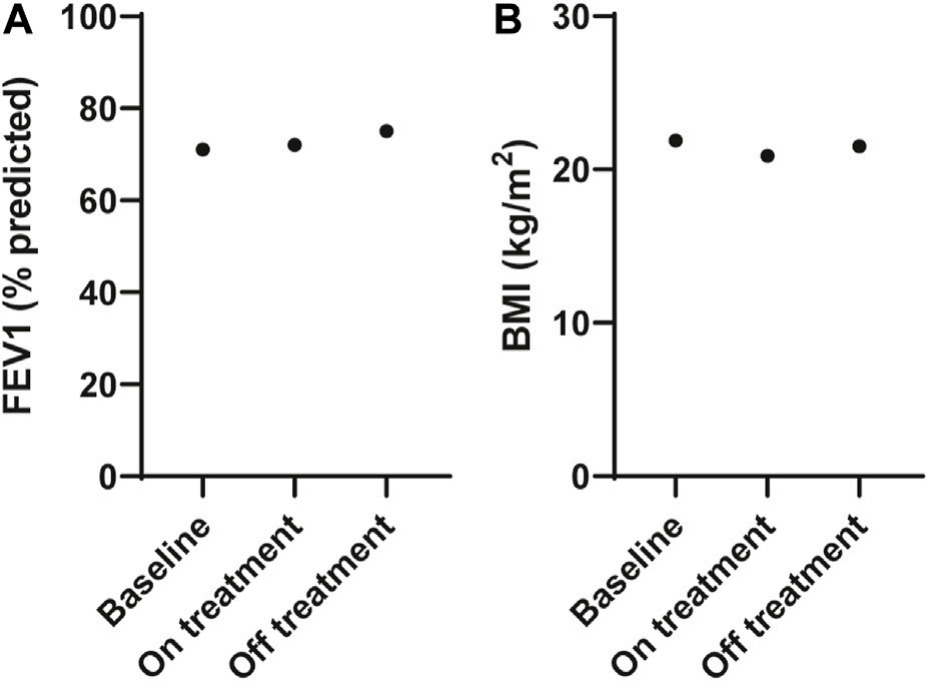
Effect of ELX/TEZ/IVA *in vivo* on lung function and body mass index in an individual with Q1291H/F508del-CFTR. **(A)** Lung function (FEV1, % predicted) and **(B)** body mass index (BMI, kg/m^2^) at baseline and on and off ELX/TEZ/IVA treatment.

The participant commenced treatment with ETI but ceased after 3 months due to adverse events that were attributed to the therapy, and no increase in FEV1 measurements [FEV1 71% predicted (2.41 L) at baseline, FEV1 72% predicted (2.42 L) on treatment]; Figure 2A; Supplementary Table S2. He had lost weight with a reduction in his BMI from 21.9 to 20.9 kg/m^2^ (Figure 2B). Extended clinical information and data for the patient can be found in Supplementary Material S1.

After considering the risks and benefits of continuing treatment it was decided to cease ETI. At follow up (3 months after ceasing ETI), all adverse symptoms had resolved his FEV1 was slightly increased at 75% predicted (2.50 L; Figure 2A) and his BMI increased to 21.5 kg/m^2^ (Figure 2B) with no ETI treatment.

### 3.2 *In silico* characterization of Q1291H-CFTR

**FIGURE 3:**
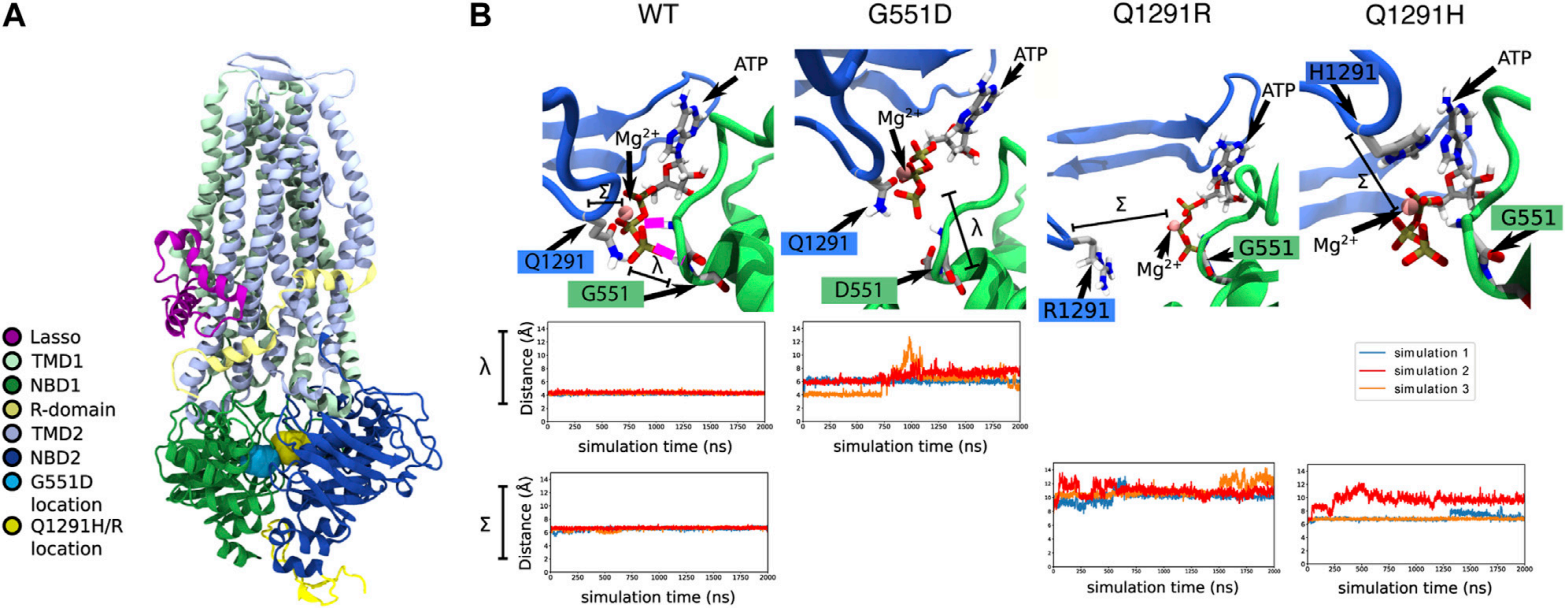
*In silico* MD simulations of G551D- and Q1291H-CFTR. **(A)** The location of the G551D and Q1291H/R mutations in CFTR, located on NBD1 and NBD2, respectively. **(B)** In WT-CFTR simulations, the G551 amino acid makes a stable hydrogen bond with the phosphate tail of an ATP + Mg^2+^ complex. In this case, 100% of our data points lie below a threshold of 5 Å, reflecting constant contact between G551 and ATP. The distance between the γ phosphate in the tail of the ATP molecule and the amide nitrogen of amino acid 551 is represented by λ. In all simulations of G551D-CFTR, this hydrogen bond with the ATP + Mg^2+^ complex is disrupted. In simulations 1 and 2 (blue and red), this disruption is immediate, while in simulation 3 (orange), the disruption occurs after roughly 700 ns. This leads to just 13% of our data points falling below a contact threshold of 5 Å. The same ATP + Mg^2+^ complex is also in proximity to amino acid 1291. The distance between alpha carbon of amino acid 1291 and the Mg^2+^ ion is represented by Σ. In simulations of WT-CFTR, the Q1291 amino acid forms a metal-coordination bond with the Mg^2+^ ion. Here, we consider amino acid 1291 to be in contact if the distance between the 1291 alpha carbon and the ion to be below 7 Å. In the case of WT-CFTR, we observe this contact to be present >99% of the time. This contact is disrupted in some simulations Q1291R and Q1291H. No simulations of Q1291R-CFTR retain close contact with Mg^2+^, with 0% of the data falling below the contact threshold of 7 Å. In Q1291H-CFTR, this interaction shows variable behavior. Simulation 1 (blue) exhibited a brief disruption of this contact after 1200 ns, while simulation 2 (red) demonstrated a disruption similar to that seen in Q1291R-CFTR. Simulation 3 (orange) displayed no perturbation from a stable contact. Overall, in Q1291H-CFTR, 57% of the simulation data displays contact between the 1291 sidechain and the nearby magnesium ion.

To determine the structural defect that may be caused in the normally spliced Q1291H isoform, Q1291H-CFTR was modelled using MD simulations. Given the proximity of Q1291 in NBD2 and G551 in NBD1—where the prototypical G551D gating mutation occurs (Bompadre et al., 2007)—to the same ATP and Mg^2+^ binding site (Figure 3A), we anticipated that Q1291H may similarly result in a CFTR gating defect. Therefore, we studied G551D alongside Q1291H and Q1291R mutations using MD simulations.

In MD simulations of WT-CFTR, G551 forms a stable hydrogen bond with the tri-phosphate tail of the bound ATP molecule. However, these contacts were consistently broken in all three simulations of G551D-CFTR (Figure 3B). Because of these fluctuations, the average distance between amino acid 551 and the ATP phosphate tail was 6.3 ± 1.2 Å in simulations of G551D-CFTR compared to 3.9 ± 0.2 Å in the WT-CFTR (distance λ in Figure 3B). These findings are consistent with the introduction of the negative charge in the aspartate (D) amino acid, which electrostatically repels the negative ATP molecule. This disruption indicates that the binding mode of ATP + Mg^2+^ seen in WT-CFTR is likely disrupted in the G551D mutant.

In simulations of WT-CFTR, the Q1291 amino acid made stable interactions with a magnesium ion. Hence, we expected that the mutation of the Q1291 amino acid would result in the disruption of ATP binding. Indeed, in simulations of Q1291R-CFTR we observed that amino acid R1291 moved away from the magnesium ion in the ATP binding site (Figure 3B). Because of these fluctuations, the average distance between amino acid 1291 and the nearby magnesium ion was 10.7 ± 0.9 Å in Q1291R-CFTR compared to 6.6 ± 0.2 Å in the WT-CFTR (distance Σ in Figure 3B). These findings are consistent with the introduction of the positive charge in the arginine (R) amino acid, which also electrostatically repels the positive magnesium ion. This indicated that, like G551D-CFTR, Q1291R-CFTR exhibited the destabilization of the native binding mode the nearby ATP + Mg^2+^ complex. The disruption of this binding site should lead to a gating defect, since proper ATP binding is required for channel opening.

In only one of the three simulations of the Q1291H mutation, disruption of the ATP + Mg^2+^ binding was observed. The average distance between the alpha carbon of amino acid 1291 and the nearby magnesium ion was 7.8 ± 1.4 Å in three simulations of Q1291H-CFTR (distance Σ in Figure 3B). In two of these simulations, however, the neutral histidine (H) sidechain was able to retain a stable contact with the magnesium ion (Figure 3B). This indicates that Q1291H may exhibit a gating defect that is less severe relative to the other mutations included in this study.

### 3.3 *In vitro* theratyping of Q1291H/F508del-CFTR in patient-derived nasal epithelial cells

**FIGURE 4:**
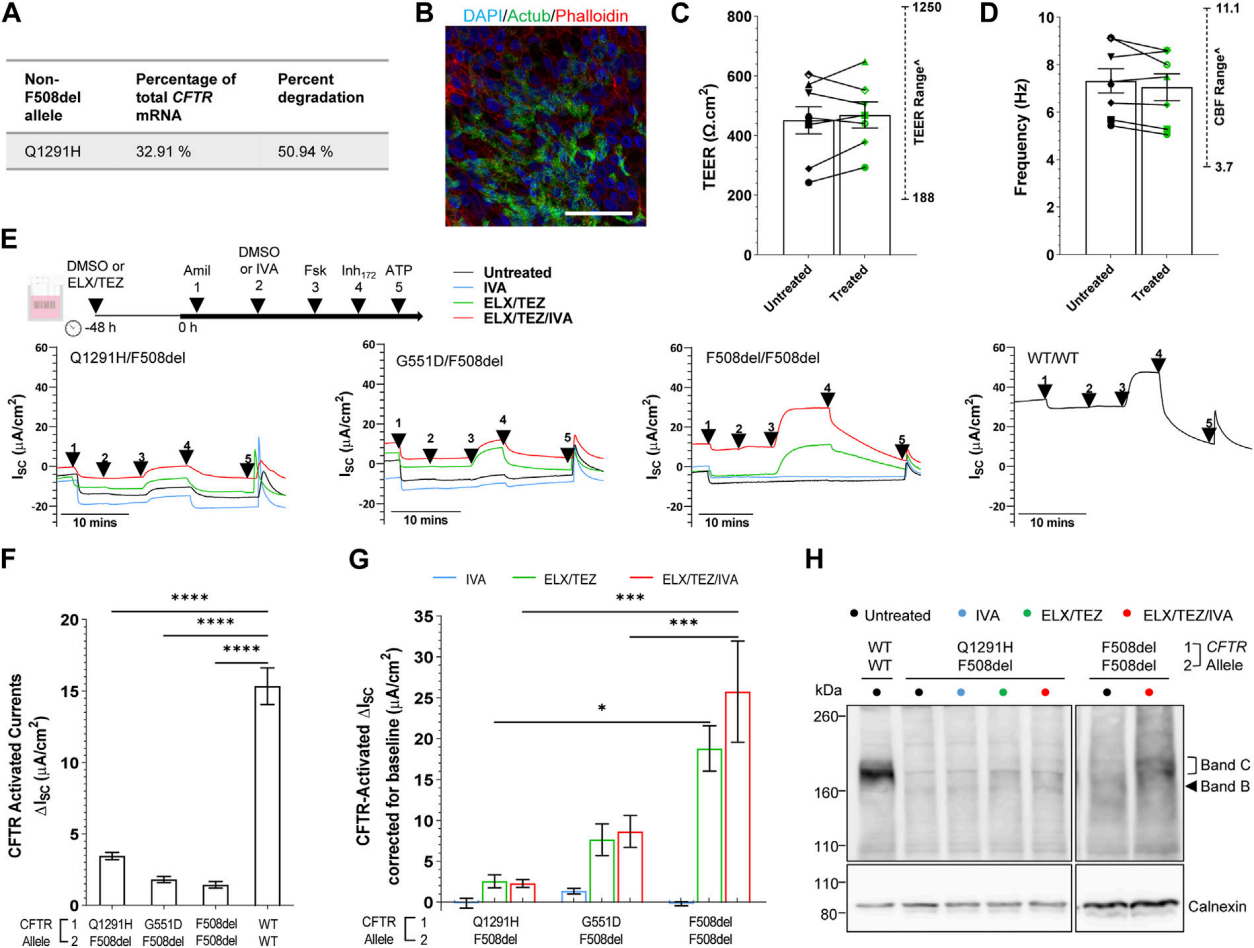
Effect of ELX/TEZ/IVA *in vitro* in Q1291H/F508del-CFTR patient-derived differentiated nasal epithelial cells. **(A)** Relative abundance and percent degradation of non-F508del (Q1291H) *CFTR* mRNA transcripts obtained with F508del primers. **(B)** Immunofluorescence staining of acetylated tubulin (Actub, green), actin (Phalloidin, red) and DAPI (blue) in Q1291H/F508del differentiated hNECs. A 63x/1.4 numerical aperture oil immersion objective was used. Scale bar: 50 µm. **(C)** Bar graph of mean transepithelial electrical resistance (TEER, Ω.cm^2^) and **(D)** cilia beat frequency (Hz) in Q1291H/F508del (*n* = 1 participant), G551D/F508del (*n* = 3 participants), F508del/F508del (*n* = 3 participants) and WT/WT (*n* = 1 participant) differentiated hNECs with and/or without ELX/TEZ treatment. Measurements for each participant were averaged from **(C)** *n* = 4–6 and **(D)** *n* = 2–3 replicate hNEC cultures and represented by a different symbol. Data are represented as Mean ± SEM. An unpaired *t*-test was used to determine statistical significance. ^The range of TEER and CBF previously reported by our lab in airway cell models at ALI (*n* = 11 non-CF, *n* = 10 CF) (Wong et al., 2022b) are indicated. **(E)** Protocol used to measure CFTR function in hNECs pre-treated at 48 h with corrector [ELX (3 μM VX-445) and TEZ (18 μM VX-661)] or untreated (0.01% DMSO), followed by sequential addition from 0 h with 100 μM apical amiloride (1. Amil), apical DMSO or IVA (10 μM VX-770) (2. DMSO or VX-770), 10 μM basal forskolin (3. Fsk), 30 μM apical CFTR-specific inhibitor (4. CFTR_Inh-172_) and 100 μM apical ATP (5. ATP). Representative Ussing chamber recordings of short circuit current (*I*_sc_) in hNECs from Q1291H/F508del, G551D/F508del, F508del/F508del and WT/WT participants **(F)** Bar graph of baseline CFTR-activated current in Q1291H/F508del (*n* = 1 participant), G551D/F508del (*n* = 3 participants), F508del/F508del (*n* = 3 participants) and WT/WT (*n* = 1 participant) differentiated hNECs following stimulation by DMSO plus Fsk. **(G)** Bar graph of CFTR-activated currents in CF participant differentiated hNECs following stimulation by IVA plus Fsk or pre-treated with ELX/TEZ and stimulated by DMSO plus Fsk or IVA plus Fsk. Measurements were corrected for baseline CFTR-activated current. Data are represented as Mean ± SEM. Each participant had *n* = 2–3 replicate hNEC cultures per treatment condition. Ordinary one-way ANOVA with Tukey’s method for multiple comparisons was used to determine statistical significance. ****p < 0.0001. **(H)** Western blot in whole cell lysates from Q1291H/F508del and WT/WT hNECs following treatment with or without IVA, ELX/TEZ or ELX/TEZ/IVA. Cell lysate of WT/WT was used as the control for CFTR band C protein size.

In patient-derived Q1291H/F508del hNECs, the relative abundance of *CFTR* mRNA was measured by allele specific qRT-PCR using primers (Clarke et al., 2019) that distinguished between presence or absence of the F508del mutation. A mean 67.09% of total *CFTR* mRNA was found to be derived from the F508del allele, with the remainder 32.91% corresponding to Q1291H *CFTR* mRNA (Figure 4A; Supplementary Table S3). We anticipated this might consist of both normally spliced Q1291H mRNA (isoform 2) and also misspliced mRNA (isoform 1) since not all PTC-bearing mRNA undergoes NMD. Assuming that mRNA is transcribed from each *CFTR* allele in a 50:50 ratio, it is approximated that 50.94% of the transcribed Q1291H *CFTR* mRNA was misspliced and degraded (isoform 1).

We next differentiated Q1291H/F508del hNECs and for comparison created differentiated hNECs from reference cell models with known and characterised CFTR defects: gating (G551D/F508del; *n* = 3), folding/maturation (F508del/F508del; *n* = 3), or no defect (WT/WT; *n* = 1; Figure 4B). Transepithelial electrical resistance (TEER) of Q1291H/F508del differentiated hNECs was 242.3 ± 24.76 Ω cm^2^ which is indicative of intact junction integrity (Brewington et al., 2018; Park et al., 2020; Awatade et al., 2021) and within the TEER range recorded for the reference cell models and that previously reported by our lab in airway cell models at ALI (188–1250 Ω cm^2^) (Wong et al., 2022b) (Figure 4C; Supplementary Table S4). Cilia marker (Actub; acetylated tubulin) was present at the apical surface of the Q1291H/F508del epithelium (Figure 4B) and functional. Cilia beat frequency (CBF) of Q1291H/F508del differentiated hNECs was 5.43 ± 0.15 Hz, within the normal physiological range of the reference cell models and that previously reported by our lab in airway cell models at ALI (3.0–11.1 Hz) (Wong et al., 2022b) (Figure 4D; Supplementary Table S4). Neither TEER nor CBF increased following ELX/TEZ treatment (Figures 4C,D).

Transepithelial ion transport was assessed via short-circuit current (*I*_sc_) in Q1291H/F508del differentiated hNECs and compared to reference differentiated hNEC models (Figure 4E; Supplementary Figures S1A,B). ENaC activity was higher in Q1291H/F508del compared to WT/WT differentiated hNECs (Supplementary Figure S1A), however this observation is not biologically unusual since CFTR is known to have regulatory action on ENaC wherein normally functioning CFTR inhibits ENaC open probability and dysfunction of CFTR activates ENaC to compensate (Gentzsch et al., 2010; Rubenstein et al., 2011). Furthermore, ENaC activity in Q1291H/F508del differentiated hNECs did not significantly differ from the CF participant control differentiated hNECs. Q1291H/F508del baseline forskolin-activated *I*_sc_ (*I*_sc-Fsk_) current did not significantly differ from G551D/F508del and F508del/F508del baseline *I*_sc-Fsk_ (Figure 4F). All CF differentiated hNECs had significantly (*p* < 0.0001) reduced *I*_sc-Fsk_ current of at most 3.45 ± 0.25 μA/cm^2^ compared to WT/WT (15.33 ± 1.28 μA/cm^2^, Figure 4F; Supplementary Table S5).

Next, we evaluated the individual components of ETI to determine their efficacy in restoring Q1291H-CFTR compared to the reference models. As expected, the potentiator IVA, which increases the channel open probability of the CFTR present at the cell surface (Yu et al., 2012), did not increase CFTR activity in F508del/F508del differentiated hNECs (0.85-fold of baseline, Figure 4G; total *I*_sc-Fsk_: 1.22 ± 0.24 μA/cm^2^, Supplementary Figure S1D; Supplementary Table S5). Consequently, IVA was unlikely to enhance F508del allele activity in either Q1291H/F508del or G551D/F508del differentiated hNECs. In G551D/F508del, potentiation with IVA resulted in a 1.78-fold increase in CFTR activity (total *I*_sc-Fsk_: 3.23 ± 0.32 μA/cm^2^; 1.42 μA/cm^2^ above baseline), which was attributed to the G551D allele potentiation. However, potentiation with IVA in the Q1291H/F508del differentiated hNECs did not increase CFTR activity above baseline (0.96-fold of baseline; total *I*_sc-Fsk_: 3.30 ± 0.60 μA/cm^2^). Thus, we concluded that Q1291H-CFTR is not responsive to potentiator rescue with IVA.

ELX and TEZ function to correct CFTR misfolding and facilitate trafficking to the cell surface, increasing the amount of CFTR available at the cell surface (Veit et al., 2020). In addition, ELX has been described to have potentiator activity (Shaughnessy et al., 2021; Veit et al., 2021). In F508del/F508del differentiated hNECs, ELX/TEZ dual therapy significantly increased CFTR activity 14.06-fold (*p* < 0.0001), reaching 18.80 μA/cm^2^ above baseline (Figure 4G; total *I*_sc-Fsk_: 20.24 ± 2.78 μA/cm^2^, Supplementary Figure S1D; Supplementary Table S5). In G551D/F508del differentiated hNECs, ELX/TEZ dual therapy resulted in a 5.27-fold increase in CFTR activity, attaining 7.72 μA/cm^2^ above baseline (total *I*_sc-Fsk_: 9.53 ± 1.95 μA/cm^2^). This enhancement is expected to be largely contributed to by restoration of the F508del allele folding defect, since the G551D mutation impairs CFTR channel opening but does not cause a folding defect (Sermet-Gaudelus, 2013). However, it is possible that the G551D allele is potentiated by ELX which has recently been shown to exhibit synergy with IVA in potentiating the G551D mutation in human airway epithelia (Shaughnessy et al., 2021; Veit et al., 2021). In contrast, ELX/TEZ dual therapy in Q1291H/F508del differentiated hNECs only increased CFTR activity by 1.74-fold, reaching only 2.55 μA/cm^2^ above baseline (total *I*_sc-Fsk_: 6.00 ± 0.80 μA/cm^2^). This increase was significantly (*p* < 0.05) lower than that observed in F508del/F508del differentiated hNECs. Consequently, the Q1291H/F508del participant had minimal CFTR rescue by ELX/TEZ, likely due to minimal restoration of their F508del allele.

We then assessed if the triple combination-ETI could restore CFTR function *in vitro* for the Q1291H-F508del participant. In comparison to the reference F508del/F508del differentiated hNECs, which demonstrated a significant 18.88-fold increase in CFTR activity with ETI treatment (*p* < 0.0001, Supplementary Figure S1D) reaching 25.75 μA/cm^2^ above baseline (Figure 4G; total *I*_sc-Fsk_: 27.19 ± 6.19 μA/cm^2^; Supplementary Table S5), the Q1291H/F508del differentiated hNECs, showed a non-significant and negligible 1.66-fold increase in CFTR activity after ETI treatment, reaching only 2.28 μA/cm^2^ above baseline (total *I*_sc-Fsk_: 5.73 ± 0.48 μA/cm^2^). In the case of the reference G551D/F508del differentiated hNECs, ETI treatment led to a 5.82-fold increase in CFTR activity, reaching 8.72 μA/cm^2^ above baseline (total *I*_sc-Fsk_: 10.53 ± 1.97 μA/cm^2^), although this increase was not statistically significant. This enhancement was 1.00 μA/cm^2^ above that achieved by ELX/TEZ dual therapy, demonstrating the additive action of ELX/TEZ and IVA. The findings indicated that the Q1291H/F508del participant experienced minimal CFTR rescue by ETI, unlike the other groups assessed.

To further investigate, we conducted western blot experiments. Mature complex-glycosylated CFTR protein (band C: ∼180 kD) was detected in the untreated F508del/F508del lysate at only 2.1% ± 1.06% of the band C in the non-CF (WT/WT) lysate (Figure 4G; Supplementary Figure S2). Following treatment with ETI, the presence of mature band C CFTR significantly (*p* < 0.01) increased to 29.36% ± 10.37% of WT/WT (Supplementary Figure S2). In contrast, Q1291H/F508del showed a low baseline presence of band C CFTR (3.18% ± 0.60% of WT/WT) and no significant change in band C CFTR expression following treatment with IVA, ELX/TEZ or ETI (5.06% ± 1.51%; 11.92% ± 1.70% and 9.90% ± 2.03% of WT/WT respectively). The low baseline presence of Q1291H/F508del mature CFTR was consistent with the electrophysiology data.

## 4 Discussion

While the most common *CFTR* mutation, F508del, is found on 70% of CF alleles, the majority of the remaining ∼ 400 identified disease-causing *CFTR* mutations are rare or ultra-rare with allelic frequencies of less than 2% (Clinical and Functional Translation of Cftr, 2023). Some of the individuals with rare mutations will be heterozygous for a well-characterized *CFTR* allele that enables them access to CFTR modulator treatment. However, as they were unlikely to have been eligible for the large-scale clinical trials there is a gap in understanding the clinical efficacy of available treatments for these heterozygous individuals. In this study, we have reported an individual with Q1291H/F508del *CFTR* genotype who became eligible for treatment with the highly effective CFTR modulator triple combination therapy, ETI. A lack of improvement in FEV1 measurements following treatment with ETI (Figure 2), along with significant adverse effects led to a conclusion that this patient was not responding positively to ETI, and treatment was ceased. Individuals with Q1291H have reduced levels of CFTR mRNA transcripts due to degradation of the misspliced product (Joynt et al., 2020). We aimed to determine the structural defect incurred by the point mutation Q1291H in the residual full-length protein. As such, *in silico* MD simulations were used to predict the structural defect resulting from the Q1291H mutation (Figure 3). Patients with a single F508del mutation in combination with a minimal function, gating or residual function *CFTR* mutation have demonstrated increases in predicted FEV1 of up to 13.8 percentage points at 4 weeks and 14.3 points at 24 weeks following ETI treatment initiation (Middleton et al., 2019; Barry et al., 2021). We therefore sought to determine if the normally spliced mRNA producing full-length CFTR protein has residual channel activity and whether it may be improved by CFTR modulator therapy. Functional and biochemical assessment of Q1291H/F508del-CFTR was performed in patient-derived cells to investigate the Q1291H mutation and the efficacy of currently approved CFTR modulators in restoring the resulting defect (Figure 4).

*In silico* modelling provides qualitative information about the structural defect occurring in full-length Q1291H-CFTR protein that is normally spliced. This helps to understand, for any CFTR that may be available at the cell surface to be targeted by modulator, which modulator may be effective in restoring the defect. MD simulations were used to investigate the consequence of Q1291H on CFTR protein structure. G551D-CFTR was used as a positive control because the mutation is known to produce a CFTR gating defect (Bompadre et al., 2007), and is in physical proximity to Q1291 except residing in NBD1 instead of NBD2. In MD simulations, G551D-CFTR exhibited broken contacts with the bound ATP molecule relative to WT-CFTR. The conformational changes observed *in silico* are also consistent with newly characterized cryo-electron microscopy structures of G551D-CFTR (Wang et al., 2022). Q1291R, another point mutation at amino acid Q1291, was similarly found to break contact with the ATP + Mg^2+^ complex, whereas Q1291H maintained a more stable bond with ATP + Mg^2+^. The decreased effect on ATP binding of the histidine mutant echoes the minor effects exerted by Q1291A found within a previous study (Berger et al., 2002). Taken together, the influence of net charge within the phosphate and Mg^2+^ binding pocket appear to outweigh other factors such as amino acid size. If true, neutral mutations such as Q1291H are thus likely to induce less severe CFTR protein dysfunction in the mature protein.

Clinical reports describe a patient with Q1291R/F508del-CFTR who is pancreatic insufficient (Dork et al., 1994), and another patient with abnormal nasal potential difference (Sermet-Gaudelus et al., 2010). Whereas two siblings with Q1291H/F508del-CFTR (one of whom is the individual in this study) were reported to be pancreatic sufficient with normal sweat chloride levels in childhood, though our individual went on to demonstrate progressive CF disease as an adult. The MD simulations and clinical reports of individuals with these *CFTR* mutations together suggest that Q1291H may result in a less severe CFTR defect and milder phenotype than Q1291R. However, phenotypic variability between patients with the same *CFTR* mutation is common and therefore we cannot draw conclusions from such small numbers of individuals. A limitation of our study is that is a *n* = 1 study and there is known to be interpatient variability in response to CFTR modulators (Boyle et al., 2014; Wainwright et al., 2015; Heijerman et al., 2019; Middleton et al., 2019). However, due to Q1291H being a rare *CFTR* mutation, the availability of patient samples is limited. We also clarify that objective measurement of *in vivo* CFTR activity was not available as no measurements of sweat chloride were conducted prior to starting ETI or whilst taking ETI therapy as it is not part of routine clinical care at the individual’s clinic. If any further CFTR modulators were trialed for this individual, routine sweat chloride testing pre- and post-treatment may be informative in addition to the usual clinical markers of treatment response (lung function, exacerbation frequency). FEV1 is a measurement of airway obstruction. As such, in a disease state—with structural damage as demonstrated by the presence of bronchiectasis—the opportunity for FEV1 improvement may be limited despite correction of the underlying CFTR defect. Furthermore, it ought to be noted that the concentration of drug in a patient’s body and as such its therapeutic effects is influenced by pharmacokinetic properties, including absorption, distribution, metabolism, and excretion (Fan and de Lannoy, 2014). Factors such as age, weight, liver function, and concomitant medications can also impact the drug’s concentration (Martinez and Amidon, 2002). As such, the concentration of modulators used in *in vitro* studies might not necessarily represent the optimal concentration for clinical use in patients and may not be directly comparable to the drug *in vivo* concentration. However, in this study, the *in vitro* results aligned with the clinical evaluation of this patient as having minimal to no response to ETI therapy.

The potentiator IVA was approved by regulatory bodies for CF patients with a G551D allele after clinical trials demonstrated significant efficacy (Ramsey et al., 2011; Davies et al., 2013). IVA stabilizes the open configuration of CFTR relative to the closed configuration (Liu et al., 2019), leading to enhanced open probability of the CFTR channel (Jih and Hwang, 2013; Yeh et al., 2019). As such, IVA compensates for the gating defect caused by G551D without directly correcting the mutation. The MD simulations in this study suggested that Q1291H-CFTR exhibits an analogous defect to G551D and Q1291R-CFTR. Hence, we might expect any normally spliced full-length Q1291H-CFTR protein to respond to IVA in a similar way to the G551D and Q1291R mutations. mRNA assessment via qPCR indicated that the Q1291H/F508del participant had approximately 50.94% misspliced transcripts undergoing degradation. This rate of missplicing is similar to that found in a previous study following creation of a Q1291H-CFTR HEK293 cell line, wherein 37.2% mRNA was reported to be normally spliced and 62.8% was misspliced (Joynt et al., 2020). Since mRNA splicing data is not informative of CFTR protein translation, folding and trafficking, biochemical and functional studies were undertaken to assess CFTR expression and functionality. We found that the high proportion of misspliced Q1291H-CFTR corresponded with an observation of reduced protein expression, as demonstrated by western blot, and minimal baseline CFTR activity as determined by ion transport electrophysiology in patient-derived Q1291H/F508del hNECs. Furthermore, a minimal response to IVA was observed (0.96-fold of baseline; total *I*_sc-Fsk_: 3.30 ± 0.60 μA/cm^2^), lower than that observed for G551D/F508del reference models. It may be that the normally spliced Q1291H is not amenable to IVA therapy or that there was insufficient normally spliced Q1291H-CFTR present at the apical membrane to be potentiated. Even still, the *in silico* modelling of Q1291H-CFTR is valuable since it provides the knowledge that Q1291H normally spliced CFTR likely incurs a gating defect. As such, future therapeutics directed at addressing the splicing defect may be effectively supplemented with a potentiator such as IVA.

The participant’s other allele, F508del, is known to incur both CFTR folding and gating defects, and as such, is expected to respond to dual corrector therapy and triple combination ETI. Restoration of the Q1291H/F508del participant’s F508del allele by corrector therapy likely accounts for the 1.74-fold increase in CFTR activity that was observed *in vitro*. Expression of mature Q1291H/F508del-CFTR protein as determined by western blot, however, was not significantly increased following corrector therapy. It is important to note that CFTR protein expression is not a direct indicator of CFTR function. As such, it is imperative to assess both CFTR protein expression and function when determining the impact of a *CFTR* mutation on CFTR, and its response to treatment. While the increase in Q1291H/F508del-CFTR activity was more discrete than the average response to corrector therapy in the G551D/F508del participants, also attributed to their single F508del allele, a variable magnitude of response was observed for the G551D/F508del participants as well as the F508del/F508del participants. A similar observation of heterogenous CFTR activation has been reported previously *in vitro* in differentiated hNECs derived from 18 patients (Pranke et al., 2019). In addition, clinical trials of patients with homozygous F508del alleles have previously shown that not all patients respond favorably to modulator treatment (Wainwright et al., 2015; Heijerman et al., 2019; Middleton et al., 2019). Hypotheses for the non-responsiveness of the F508del allele to ETI include the presence of a cis variant in complex allele with F508del, which has been shown not rescuable by CFTR modulator combinations LUM/IVA or ELX/TEZ *in vitro* (Baatallah et al., 2018; Sondo et al., 2022). Furthermore, no changes in outcomes were observed for a six-year-old patient carrying the complex allele F508del; L467F with F508del (Terlizzi et al., 2022). For patients with a F508del allele who do not respond to modulator therapy *in vitro*, such as the patient in this study, screening for cis variants may be helpful in informing clinical decisions regarding a patient’s treatment.

In contrast to the lack of CFTR activation in response to ETI for our Q1291H/F508del participant [2.28 μA/cm^2^ increase from baseline (1.66-fold)], CFBE cells expressing Q1291H cDNA have previously been shown to have an approximate two-fold increase in CFTR activity in response to combination treatment with LUM/IVA (Joynt et al., 2020). A possible explanation may be that CFTR modulator efficacy is cell-type dependent (Clancy et al., 2019; Tomati et al., 2022). Furthermore, a limitation of cDNA-based systems is that they contain only the coding sequence and not any intronic sequences or regulatory elements. As such, they only enable assessment of protein processing and function, and may overlook RNA-level effects such as missplicing. In comparison, expression minigene (EMG) systems include the intronic sequences necessary for protein expression and function and are therefore an important tool for studying regulation of mRNA splicing (Cooper, 2005) and have been shown to be representative on *in vivo* mRNA splicing (Masvidal et al., 2014; Sharma et al., 2014; Sharma et al., 2018). The importance of using an appropriate model system when assessing splicing variants is highlighted by the report that cDNA bearing c.523A>G produces functional protein, while an EMG bearing the same variant produces no functional protein (Joynt et al., 2020). This emphasizes the possibility of misrepresenting modulator response when relying on cDNA-based systems. While Joynt et al. assist in understanding the Q1291H protein defect and modulator response that would result in correctly spliced Q1291H protein, the modulator response is likely overrepresented since the misspliced product is not accounted for. Relying on a cDNA-based system could be misleading and not reflective of the response that would be observed *in vivo*. In line with this, it has been reported previously that the activity of CFTR corrector compounds in cell lines used for heterologous expression are not predictive of their efficacy in primary airway epithelial cells of CF patients (Pedemonte et al., 2010), and that modulator response may be overestimated in recombinant cell expression systems (Sondo et al., 2011). As such, testing modulator response in primary patient cells is important, especially for the Q1291H mutation since it has varying clinical consequences due to missplicing (Joynt et al., 2020).

The absence of heterozygous F508del controls in the allele-specific qPCR assay poses a limitation in interpreting our results. Consequently, the presence of a putative shorter splicing variant and the potential impact of a PTC on CFTR degradation remain inconclusive. Previous work by Clarke et al. has validated the primers used in our assay for several CFTR mutations, including heterozygous mutations, which offers some confidence in our methodology (Clarke et al., 2019). Nevertheless, to obtain more definitive conclusions, future studies should consider employing complementary sequencing techniques to confirm the presence and the precise nature of the splicing variants. This would help to determine the amount of NMD, which has been shown to be responsible for degradation of up to 80% of PTC-bearing mRNAs, yet with variation among both different PTC mutations and individuals with the same genotype (Clarke et al., 2019). While we did not explore the degree of mRNA NMD, it is important to note that investigating the activation of putative shorter versions by modulators could also shed light on the underlying mechanisms and potential therapeutic approaches for individuals with similar genetic profiles. For mutations at the C terminal of CFTR, it is possible that such a late occurrence of a PTC within the *CFTR* sequence may result in expression of a truncated CFTR product that may remain amenable to CFTR modulator treatment. However, while human nasal cells of homozygous W1282X-CFTR cells (Mutyam et al., 2017) exhibited partial activity in the truncated state, and improved CFTR function in the presence of IVA, our participant did not. It was reasoned that this was due to W1282X-CFTR retaining partial chloride channel function, even in its truncated form, when expressed at the cell surface in sufficient quantity. In contrast, IVA was shown to have negligible effects on cells expressing R1162X-CFTR (Mutyam et al., 2017) which does not have function at the cell surface in the truncated state (Rowe et al., 2007). Since we observed low Q1291H-CFTR surface expression *via* Western blot (3.18% ± 0.60% of WT/WT) and low CFTR activity via ion transport electrophysiology (3.45 ± 0.25 μA/cm^2^), we suggest that similar to the previous report of R1162X-CFTR, Q1291H-CFTR was non-responsive to IVA due to there being an insufficient quantity of protein expressed at the cell surface, hence unavailable to be targeted by IVA.

Although a larger number of technical replicas would be advantageous for validation and extension of our findings, the electrophysiological data is limited due to the constraints posed by working with low passage (P1/P2) patient-derived human nasal cells. Despite this limitation, our findings contribute to an understanding of the underlying biological processes and pave the way for future research. We encourage subsequent studies to build upon our preliminary findings using larger sample sizes and alternative methodologies to further substantiate and refine the observed trends, enabling a more quantitative analysis. Overall, our results demonstrated that Q1291H/F508del CFTR resulted in reduced baseline CFTR activity, and that this individual had minimal response to IVA, ELX/TEZ and ETI therapy *in vitro.* Variability in the clinical presentation of CF and response to modulators highlight a need for personalized drug response testing as it is known that drug response is heterogenous between different individuals, even those of the same mutation.

## Data Availability

The raw data supporting the conclusion of this article will be made available by the authors, without undue reservation.
