# Supplementary material for: Q1291H-CFTR molecular dynamics simulations and ex vivo theratyping in nasal epithelial models and clinical response to elexacaftor/tezacaftor/ivacaftor in a Q1291H/F508del patient
Source: Front Mol Biosci. 2023 Jun 1;10:1148501. doi: 10.3389/fmolb.2023.1148501 (PMC10267335; doi:10.3389/fmolb.2023.1148501)
Supplement: Supplementary file 1 [file Table1.DOCX]

Supplementary Material

## Supplementary Material 1. Extended clinical history and treatment response

We report the case of a 56-year-old individual with CF with the Q1291H/F508del *CFTR* genotype. The participant presented with severe asthma at age six years but with no pancreatic disorders and a sweat test result which was normal cystic fibrosis was considered unlikely. He continued to be managed as an asthmatic. By adulthood he had significant impairment in FEV1, bronchiectatic changes on his chest x-ray and azoospermia had been identified. CF complications included lower airway colonization with staph aureus, aspergillus fumigatus and Mycobacterium abscessus (participant elected not to undergo eradication treatment), as well as CF sinus disease, with polypectomy and debridement required at age 54 years. His most recent chest imaging demonstrated stable upper lobe predominant bronchiectasis. His asthma was controlled using medium dose ICS/LABA combination. He was a lifelong nonsmoker, pancreatic sufficient and had no gastrointestinal manifestation of CF with a normal screening colonoscopy in 2019. He had an incidental finding of Anti-PM/Scl antibodies identified during CF annual review investigations which included extended autoimmune screening. He had no associated clinical phenotype for this antibody and normal CK levels.

The participant commenced treatment with ETI but ceased after three months due to adverse events that were attributed to the therapy. The adverse events included a rash, significant adverse changes in bowel habit and a single episode of shortness of breath, dizziness and profound muscle weakness after sudden exertion (running to catch a train) which occurred six weeks after commencing treatment with ETI and resolved after resting at home. Hypertension (blood pressure 144/90) was identified at a primary care review (GP) following this event. Investigations identified a raised average systolic blood pressure (BP) (130/89; previously measured as 120/80 prior to commencing treatment with ETI). Nocturnal leg cramps developed, and a previous essential tremor worsened, causing difficulty with fine motor control. Serum CMP and EUC levels were within normal ranges.

OGTT 1 year prior to ETI treatment

|  | 0 min | 60 min | 120 min |
| --- | --- | --- | --- |
| Insulin | 24 | 204 | 264 |
| Glucose | 4.9 | 9.9 | 6.5 |

OGTT after 1 month of ETI treatment

|  | 0 min | 60 min | 120 min |
| --- | --- | --- | --- |
| Insulin | 18 | 138 | 144 |
| Glucose | 5 | 11.4 | 6.7 |

At review three months after starting ETI treatment he had lost weight with a reduction in his BMI from 21.9 to 20.9 Kg/m^2^. He reported mildly reduced sputum load only. After considering the risks and benefits of continuing treatment it was decided to cease ETI. At follow up three months after ceasing therapy, the patient had no further episodes of rash or muscle weakness and all gastrointestinal side effects and his tremor had ceased. This supported the initial assessment of his symptoms as ETI side effects rather than the onset of disease symptoms related to his yet asymptomatic positive Anti-PM/Scl status.


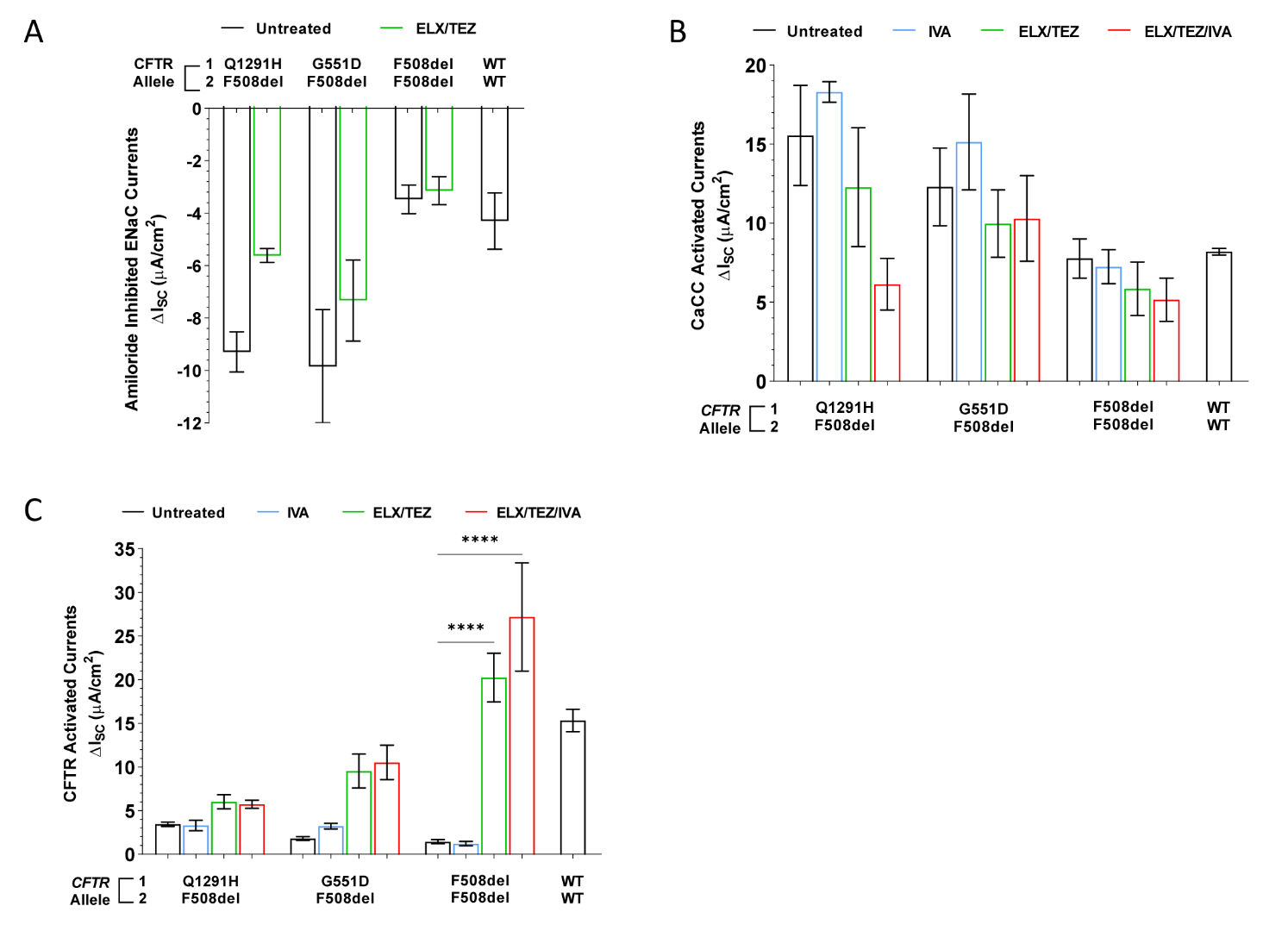


**Supplementary Figure 1. Effect of ELX/TEZ/IVA on short circuit currents in Q1291H/F508del-CFTR patient-derived differentiated nasal epithelial cells.** Bar graphs of mean **(A)** amiloride-inhibited epithelial sodium channel (ENaC) currents, **(B)** ATP-activated calcium-dependent chloride channel (CaCC) currents and **(C)** CFTR-activated currents. Q1291H/F508del currents are compared to G551D/F508del (n=3 participants), F508del/F508del (n=3 participants) and WT/WT (n=1 participant) currents. Data represents mean current in polarized cultures pre-treated with ELX/TEZ or DMSO and stimulated by IVA or DMSO plus Fsk. Data are represented as Mean ± SEM. Each participant had n=2-3 replicate hNEC cultures per treatment condition. Participants are represented by different symbols. To determine statistical significance, Kruskal-Wallis test was used for non-parametric data (A-C) and ordinary one-way ANOVA with Tukey’s method for multiple comparisons was used for parametric data (D). ****p*<0.001, *****p*<0.0001. Refer to Supplementary Table 5 for short-circuit current values.


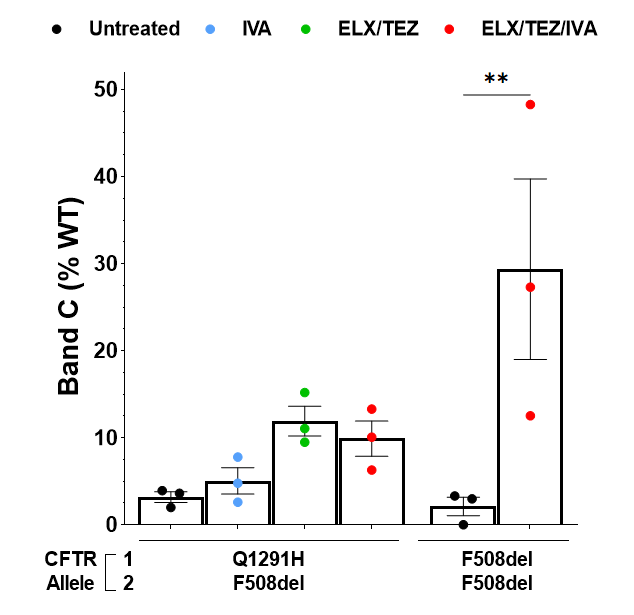


**Supplementary Figure 2. Effect of ELX/TEZ/IVA** **on Q1291H/F508del-CFTR maturation in patient-derived differentiated nasal epithelial cells.** Bar graphs of band C CFTR protein expression expressed as a percentage of WT/WT band C CFTR. Measurements were from western blots of whole cell lysates from Q1291H/F508del, F508del/F508del and WT/WT hNECs following treatment with or without IVA, ELX/TEZ or ELX/TEZ/IVA. Band C represents mature, complex-glycosylated CFTR. Densitometry was normalized to the calnexin loading control. Technical replicates of the western blot were performed (n=3) and indicated by individual data points. Data are represented as Mean ± SEM. Ordinary one-way ANOVA with Tukey’s method for multiple comparisons was used to determine statistical significance. ***p*<0.01.

## Supplementary Tables

**Supplementary Table 1. Details of study participants.**

| Participant ID | *CFTR* genotype | Age (yr) |
| --- | --- | --- |
| CF1 | Q1291H/F508del | 56 |
| CF2 | G551D/F508del | 0 |
| CF3 | G551D/F508del | 9 |
| CF4 | G551D/F508del | 5 |
| CF6 | F508del/F508del | 1 |
| CF7 | F508del/F508del | 2 |
| CF8 | F508del/F508del | 3 |
| Non-CF | WT/WT | 1 |

**Supplementary Table 2. Full spirometry data for individual with Q1291H/F508del-CFTR.**

|  | Parameters | LLN | Pred | ULN | Actual | %Pred |
| --- | --- | --- | --- | --- | --- | --- |
| May 2022 | FEV1 (L) | 2.57 | 3.35 | 4.09 | 2.41 | 71 |
|  | FVC (L) | 3.28 | 4.25 | 5.23 | 4.24 | 99 |
|  | FEV1/FVC (%) | 67 | 79 | 89 | 57 | 71 |
|  | FEF Max (L/Sec) | 6.67 | 8.76 | 10.85 | 7.22 | 82 |
|  | FEF 25-75% (L/Sec) | 1.54 | 3.00 | 4.95 | 1.05 | 35 |
| August 2022 | FEV1 (L) | 2.54 | 3.32 | 4.06 | 2.42 | 72 |
|  | FVC (L) | 3.25 | 4.22 | 5.19 | 4.32 | 102 |
|  | FEV1/FVC (%) | 67 | 79 | 89 | 56 | 71 |
|  | FEF Max (L/Sec) |  | 8.70 |  | 8.71 | 100 |
|  | FEF 25-75% (L/Sec) | 1.51 | 2.95 | 4.88 | 1.01 | 34 |

**Supplementary Table 3. Data for the qPCR performed in patient-derived nasal epithelial cells.**

|  |  | Q1291H/ F508del | F508del/ F508del | F508del/ F508del | F508del/ F508del | No RT | No template |
| --- | --- | --- | --- | --- | --- | --- | --- |
| Average Ct | non-F508del | 32.15 | 33.84 | 33.64 | 32.88 | 36.81 | 38.04 |
|  | F508del | 30.78 | 33.79 | 33.14 | 32.40 | 37.76 | 36.72 |
|  | ACTB | 20.40 | 20.15 | 18.75 | 18.88 | 36.27 | > 40 |
| ΔCt | non-F508del | 11.75 | 13.69 | 14.89 | 14.00 |  |  |
|  | F508del | 10.38 | 13.64 | 14.39 | 13.52 |  |  |
| ΔΔCt | non-F508del | -2.44 |  |  |  |  |  |
|  | F508del | -3.47 |  |  |  |  |  |
| FC = 2^(-ΔΔCT) | non-F508del | 5.44 |  |  |  |  |  |
|  | F508del | 11.08 |  |  |  |  |  |
| Total *CFTR* mRNA (FC non-F508del + FC F508del) |  | 16.52 |  |  |  |  |  |
| % F508del mRNA |  | 67.09 |  |  |  |  |  |
| % non-F508del mRNA |  | 32.91 |  |  |  |  |  |
| % Degradation of non-F508del transcripts |  | 50.94 |  |  |  |  |  |

Data presented are the calculations for relative mRNA abundance and degradation. As described previously (1), products were quantified using the ΔΔCT method. To calculate the ΔCT, mRNA expression was normalized using the ACTB reference gene. To calculate the ΔΔCT, the ΔCT was normalized to the mean ΔCT of the calibrator samples (n=3 F508del/F508del). The fold change (FC) of F508del and non-F508del products was calculated using the formula FC = 2(–ΔΔCT). Abundance of mRNA transcripts derived from each allele was expressed as a percentage of total *CFTR* mRNA. To calculate the percent degradation of non-F508del transcripts, the following formula was used: % degradation = ((Y–X)/Y) × 100, where X and Y are percent CFTR transcript derived from non-F508del and F508del alleles, respectively. For further information see Method section 2.4.

**Supplementary Table 4. Data for the transepithelial electrical resistance in patient-derived differentiated nasal epithelial cells.**

| Participant ID | TEER | | CBF | |
| --- | --- | --- | --- | --- |
|  | Untreated | Treated | Untreated | Treated |
| CF1 | 242.3 ± 24.76 | 292.5 ± 14.43 | 5.43 ± 0.15 | 5.06 ± 0.08 |
| CF2 | 436.5 ± 46.65 | 467.7 ± 26.11 | 5.68 ± 0.02 | 5.29 ± 0.02 |
| CF3 | 571.3 ± 21.12 | 648.0 ± 34.66 | 7.30 ± 0.42 | 7.50 ± 0.21 |
| CF4 | 288.7 ± 24.05 | 378.3 ± 19.70 | 6.39 ± 0.18 | 6.31 ± 0.10 |
| CF6 | 459.3 ± 21.82 | 440.0 ± 72.76 | 9.13 ± 0.68 | 8.00 ± 0.24 |
| CF7 | 604.5 ± 47.56 | 552.0 52.00 | 9.12 ± 0.12 | 8.60 ± 0.02 |
| CF8 | 542.5 ± 41.06 | 503.0 ± 35.82 | 8.33 ± 0.08 | 8.58 ± 0.19 |
| Non-CF | 464.7 ± 23.13 | n/a | 7.17 ±0.39 | n/a |

Data presented are transepithelial electrical resistance (TEER) and cilia beat frequency (CBF) measurements for each participant as in Figure 4C-D. Data are represented as Mean ± SEM.

**Supplementary Table 5. Data for the short-circuit current in patient-derived differentiated nasal epithelial cells.**

|  |  | ΔAmil | ΔATP | ΔCFTR-Activated |
| --- | --- | --- | --- | --- |
| Q1291H/F508del (n=1 participant) | DMSO | -9.29 ± 0.76 | 15.56 ± 3.17 | 3.45 ± 0.25 |
|  | IVA | n/a | 18.31 ± 0.66 | 3.30 ± 0.60 |
|  | ELX/TEZ | -5.61 ± 0.27 | 12.28 ± 3.76 | 6.00 ± 0.80 |
|  | ELX/TEZ/  IVA | n/a | 6.14 ± 1.64 | 5.73 ± 0.48 |
| G551D/F508del (n=3 participants) | DMSO | -9.85 ± 2.17 | 12.30 ± 2.46 | 1.81 ± 0.21 |
|  | IVA | n/a | 15.14 ± 3.03 | 3.23 ± 0.32 |
|  | ELX/TEZ | -7.33 ± 1.54 | 9.98 ± 2.13 | 9.53 ± 1.95 |
|  | ELX/TEZ/  IVA | n/a | 10.30 ± 2.71 | 10.53 ± 1.97 |
| F508del/F508del (n=3 participants) | DMSO | -3.47 ± 0.54 | 7.77 ± 1.24 | 1.44 ± 0.24 |
|  | IVA | n/a | 7.25 ± 1.08 | 1.22 ± 0.24 |
|  | ELX/TEZ | -3.14 ± 0.54 | 5.85 ± 1.69 | 20.24 ± 2.78 |
|  | ELX/TEZ/  IVA | n/a | 5.16 ± 1.36 | 27.19 ± 6.19 |
| WT/WT  (n=1 participant) | DMSO | -4.30 ± 1.08 | 8.20 ± 0.21 | 15.33 ± 1.28 |

Data presented are short-circuit current values for amiloride inhibited ENaC currents (ΔAmil), ATP-activated currents (ΔATP) and CFTR-activated currents (ΔCFTR-Activated). Data are represented as Mean ± SEM.

**References**

1. Clarke LA, Awatade NT, Felicio VM, Silva IA, Calucho M, Pereira L, et al. The Effect of Premature Termination Codon Mutations on Cftr Mrna Abundance in Human Nasal Epithelium and Intestinal Organoids: A Basis for Read-through Therapies in Cystic Fibrosis. *Hum Mutat* (2019) 40(3):326-34. Epub 2018/11/30. doi: 10.1002/humu.23692.
